# 2044 Investigation of patient-reported outcomes following ACL reconstruction using Rasch analysis

**DOI:** 10.1017/cts.2018.88

**Published:** 2018-11-21

**Authors:** Jenny Hunnicutt, Chris Gregory, Brian Pietrosimone, Chris Kuenze, Brittany Hand, Craig Velozo

**Affiliations:** Medical University of South Carolina

## Abstract

OBJECTIVES/SPECIFIC AIMS: The knee injury osteoarthritis and outcomes survey (KOOS) is a commonly used instrument to measure patient-reported quality of life (QOL) post-ACLR. The purpose is to evaluate the psychometric properties of the QOL subscale of the KOOS. METHODS/STUDY POPULATION: Rasch analysis of KOOS QOL subscale from 39 individuals 1–2 years post ACLR was conducted. Measurement properties and model fit of the rating scale, items, and persons were evaluated. Relationship of item difficulties and person measures was evaluated using probability curves and item maps. Reliability indicators were also examined. RESULTS/ANTICIPATED RESULTS: All items demonstrated infit and outfit mean squares and standard *z*-scores. The majority of persons (n=38, 97.4%) demonstrated fit to the Rasch model. However, ceiling effects were noted (n=4, 10.26%), indicating some participants report higher QOL than is measurable. The mean person measure was 1.73 logits higher than the mean item measure: this sample is skewed toward higher QOL. Person reliability was adequate (0.67) and person separation was 1.42. Calculation of person strata revealed that the KOOS QOL separated participants into 2 strata. DISCUSSION/SIGNIFICANCE OF IMPACT: Although all items of the KOOS QOL fit the model, not all categories of the rating scale were used. Overall, this sample reported high QOL, which is to be expected given the time since ACLR. If participants with a broader range of time since ACLR were included, that the KOOS QOL could identify additional person strata.

